# Supplementary material for: Comparative genomics provides new insights into the diversity, physiology, and sexuality of the only industrially exploited tremellomycete: Phaffia rhodozyma
Source: BMC Genomics. 2016 Nov 9;17:901. doi: 10.1186/s12864-016-3244-7 (PMC5103461; doi:10.1186/s12864-016-3244-7)
Supplement: Additional file 6: — List of orphan genes with links to PFAM (related to Additional file 1: Table S1). (ZIP 1428 kb) [file 12864_2016_3244_MOESM6_ESM.zip › BLAST_HTML_FTR/G03980_P.html]

BLAST Search Results


```
BLASTP 2.2.27+


Reference:
Stephen F. Altschul, Thomas L. Madden, Alejandro A. Schäffer,
Jinghui Zhang, Zheng Zhang, Webb Miller, and David J. Lipman (1997),
"Gapped BLAST and PSI-BLAST: a new generation of protein database
search programs", Nucleic Acids Res. 25:3389-3402.


Reference for
composition-based statistics:
Alejandro A. Schäffer, L. Aravind, Thomas L. Madden, Sergei
Shavirin, John L. Spouge, Yuri I. Wolf, Eugene V. Koonin, and
Stephen F. Altschul (2001), "Improving the accuracy of PSI-BLAST
protein database searches with composition-based statistics and
other refinements", Nucleic Acids Res. 29:2994-3005.


Database: nr
           71,551,133 sequences; 26,053,659,533 total letters


Query= G03980_P

Length=283
                                                                      Score     E
Sequences producing significant alignments:                          (Bits)  Value

emb|CDZ97939.1|  hypothetical protein [Xanthophyllomyces dendrorh...   574    0.0  
ref|WP_041743304.1|  phospho-2-dehydro-3-deoxyheptonate aldolase ...  38.1    8.6  
ref|WP_018136386.1|  phospho-2-dehydro-3-deoxyheptonate aldolase ...  38.1    8.6  
ref|WP_009651129.1|  phospho-2-dehydro-3-deoxyheptonate aldolase ...  38.1    8.9  


 >emb|CDZ97939.1| hypothetical protein [Xanthophyllomyces dendrorhous]
Length=282

 Score =  574 bits (1480),  Expect = 0.0, Method: Compositional matrix adjust.
 Identities = 282/282 (100%), Positives = 282/282 (100%), Gaps = 0/282 (0%)

Query  1    MVKAHVITWSSTPHLPGSFHHDPPADEQEPDEYTRAQDVRCLVPTEIVWLMGLAGFILSI  60
            MVKAHVITWSSTPHLPGSFHHDPPADEQEPDEYTRAQDVRCLVPTEIVWLMGLAGFILSI
Sbjct  1    MVKAHVITWSSTPHLPGSFHHDPPADEQEPDEYTRAQDVRCLVPTEIVWLMGLAGFILSI  60

Query  61   LAIVTPLRCYIQPCYTNKTVPMTPSYYFARNRPFFPQRPASPHRRPSSASTMSSHSSTST  120
            LAIVTPLRCYIQPCYTNKTVPMTPSYYFARNRPFFPQRPASPHRRPSSASTMSSHSSTST
Sbjct  61   LAIVTPLRCYIQPCYTNKTVPMTPSYYFARNRPFFPQRPASPHRRPSSASTMSSHSSTST  120

Query  121  ASSTLLDTPNSESSLQSILIVSHPGPSTDREMLPPTKRVSFKEDLVQQELDVTFRMERPK  180
            ASSTLLDTPNSESSLQSILIVSHPGPSTDREMLPPTKRVSFKEDLVQQELDVTFRMERPK
Sbjct  121  ASSTLLDTPNSESSLQSILIVSHPGPSTDREMLPPTKRVSFKEDLVQQELDVTFRMERPK  180

Query  181  PPAAPVDVWALAVMDDLIVLEESHTEEFSRANAQKRNRLTKTLSFSARCPMTSQSPPRLS  240
            PPAAPVDVWALAVMDDLIVLEESHTEEFSRANAQKRNRLTKTLSFSARCPMTSQSPPRLS
Sbjct  181  PPAAPVDVWALAVMDDLIVLEESHTEEFSRANAQKRNRLTKTLSFSARCPMTSQSPPRLS  240

Query  241  PTPSFSSTMSDPSSNSLHQTSEESLSRGRQTRKNKLFRKRSN  282
            PTPSFSSTMSDPSSNSLHQTSEESLSRGRQTRKNKLFRKRSN
Sbjct  241  PTPSFSSTMSDPSSNSLHQTSEESLSRGRQTRKNKLFRKRSN  282


>ref|WP_041743304.1| phospho-2-dehydro-3-deoxyheptonate aldolase [Campylobacter curvus]
Length=446

 Score = 38.1 bits (87),  Expect = 8.6, Method: Compositional matrix adjust.
 Identities = 26/78 (33%), Positives = 36/78 (46%), Gaps = 12/78 (15%)

Query  207  EFSRANAQKRNRLTKTLSFSARCPMTSQSPPRLSPTPSFSS------------TMSDPSS  254
            E S   A+  + LTKTLSF   C +TSQ+ P L+ T  ++S            T  D  +
Sbjct  192  EISEKYAKLADELTKTLSFMEACGITSQNTPTLNQTAVYTSHEALLLPYEEAMTRQDSLT  251

Query  255  NSLHQTSEESLSRGRQTR  272
            N  +  S   L  G +TR
Sbjct  252  NDWYDCSAHMLWIGERTR  269


>ref|WP_018136386.1| phospho-2-dehydro-3-deoxyheptonate aldolase [Campylobacter curvus]
Length=446

 Score = 38.1 bits (87),  Expect = 8.6, Method: Compositional matrix adjust.
 Identities = 26/78 (33%), Positives = 36/78 (46%), Gaps = 12/78 (15%)

Query  207  EFSRANAQKRNRLTKTLSFSARCPMTSQSPPRLSPTPSFSS------------TMSDPSS  254
            E S   A+  + LTKTLSF   C +TSQ+ P L+ T  ++S            T  D  +
Sbjct  192  EISEKYAKLADELTKTLSFMEACGITSQNTPTLNQTAVYTSHEALLLPYEEAMTRQDSLT  251

Query  255  NSLHQTSEESLSRGRQTR  272
            N  +  S   L  G +TR
Sbjct  252  NDWYDCSAHMLWIGERTR  269


>ref|WP_009651129.1| phospho-2-dehydro-3-deoxyheptonate aldolase [Campylobacter sp. 
FOBRC14]
 gb|EJP74217.1| 3-deoxy-7-phosphoheptulonate synthase [Campylobacter sp. FOBRC14]
Length=446

 Score = 38.1 bits (87),  Expect = 8.9, Method: Compositional matrix adjust.
 Identities = 26/78 (33%), Positives = 36/78 (46%), Gaps = 12/78 (15%)

Query  207  EFSRANAQKRNRLTKTLSFSARCPMTSQSPPRLSPTPSFSS------------TMSDPSS  254
            E S   A+  + LTKTLSF   C +TSQ+ P L+ T  ++S            T  D  +
Sbjct  192  EISEKYAKLADELTKTLSFMEACGITSQNTPTLNQTAVYTSHEALLLPYEEAMTRQDSLT  251

Query  255  NSLHQTSEESLSRGRQTR  272
            N  +  S   L  G +TR
Sbjct  252  NDWYDCSAHMLWIGERTR  269


Lambda      K        H        a         alpha
   0.317    0.128    0.384    0.792     4.96 

Gapped
Lambda      K        H        a         alpha    sigma
   0.267   0.0410    0.140     1.90     42.6     43.6 

Effective search space used: 2062600455944


  Database: nr
    Posted date:  Sep 23, 2015 12:05 AM
  Number of letters in database: 26,053,659,533
  Number of sequences in database:  71,551,133


Matrix: BLOSUM62
Gap Penalties: Existence: 11, Extension: 1
Neighboring words threshold: 11
Window for multiple hits: 40
```
